# Genome Update of the Dimorphic Human Pathogenic Fungi Causing Paracoccidioidomycosis

**DOI:** 10.1371/journal.pntd.0003348

**Published:** 2014-12-04

**Authors:** José F. Muñoz, Juan E. Gallo, Elizabeth Misas, Margaret Priest, Alma Imamovic, Sarah Young, Qiandong Zeng, Oliver K. Clay, Juan G. McEwen, Christina A. Cuomo

**Affiliations:** 1 Cellular and Molecular Biology Unit, Corporación para Investigaciones Biológicas, Medellín, Colombia; 2 Institute of Biology, Universidad de Antioquia, Medellín, Colombia; 3 Doctoral Program in Biomedical Sciences, Universidad del Rosario, Bogotá, Colombia; 4 Broad Institute of MIT and Harvard, Cambridge, Massachusetts, United States of America; 5 School of Medicine and Health Sciences, Universidad del Rosario, Bogotá, Colombia; 6 School of Medicine, Universidad de Antioquia, Medellín, Colombia; University of California, San Diego, School of Medicine, United States of America

## Abstract

Paracoccidiodomycosis (PCM) is a clinically important fungal disease that can acquire serious systemic forms and is caused by the thermodimorphic fungal *Paracoccidioides* spp. PCM is a tropical disease that is endemic in Latin America, where up to ten million people are infected; 80% of reported cases occur in Brazil, followed by Colombia and Venezuela. To enable genomic studies and to better characterize the pathogenesis of this dimorphic fungus, two reference strains of *P. brasiliensis* (Pb03, Pb18) and one strain of *P. lutzii* (Pb01) were sequenced [1]. While the initial draft assemblies were accurate in large scale structure and had high overall base quality, the sequences had frequent small scale defects such as poor quality stretches, unknown bases (N's), and artifactual deletions or nucleotide duplications, all of which caused larger scale errors in predicted gene structures. Since assembly consensus errors can now be addressed using next generation sequencing (NGS) in combination with recent methods allowing systematic assembly improvement, we re-sequenced the three reference strains of *Paracoccidioides* spp. using Illumina technology. We utilized the high sequencing depth to re-evaluate and improve the original assemblies generated from Sanger sequence reads, and obtained more complete and accurate reference assemblies. The new assemblies led to improved transcript predictions for the vast majority of genes of these reference strains, and often substantially corrected gene structures. These include several genes that are central to virulence or expressed during the pathogenic yeast stage in *Paracoccidioides* and other fungi, such as *HSP90*, *RYP1-3*, *BAD1*, catalase B, alpha-1,3-glucan synthase and the beta glucan synthase target gene *FKS1*. The improvement and validation of these reference sequences will now allow more accurate genome-based analyses. To our knowledge, this is one of the first reports of a fully automated and quality-assessed upgrade of a genome assembly and annotation for a non-model fungus.

## Author Summary

The fungal genus *Paracoccidioides* is the causal agent of paracoccidioidomycosis (PCM), a neglected tropical disease that is endemic in several countries of South America. *Paracoccidioides* is a pathogenic dimorphic fungus that is capable of converting to a virulent yeast form after inhalation by the host. Therefore the molecular biology of the switch to the yeast phase is of particular interest for understanding the virulence of this and other human pathogenic fungi, and ultimately for reducing the morbidity and mortality caused by such fungal infections. We here present the strategy and methods we used to update and improve accuracy of three reference genome sequences of *Paracoccidioides* spp. utilizing state-of-the-art Illumina re-sequencing, assembly improvement, re-annotation, and quality assessment. The resulting improved genome resource should be of wide use not solely for advancing research on the genetics and molecular biology of *Paracoccidioides* and the closely related pathogenic species *Histoplasma* and *Blastomyces*, but also for fungal diagnostics based on sequencing or molecular assays, characterizing rapidly changing proteins that may be involved in virulence, SNP-based population analyses and other tasks that require high sequence accuracy. The genome update and underlying strategy and methods also serve as a proof of principle that could encourage similar improvements of other draft genomes.

## Introduction

*Paracoccidioides* spp. is a thermally dimorphic pathogenic fungus that causes paracoccidioidomycosis (PCM), a neglected health-threatening human systemic mycosis endemic to Latin America where up to ten million people are infected. Disease can progress slowly, with roughly five new cases of disease per million infected individuals per year, with a male to female ratio of 13 to 1. About 80% of PCM cases occur in Brazil, followed by Colombia and Venezuela [2].

Within the *Paracoccidioides* genus, the three characterized phylogenetic lineages of *P. brasiliensis* (PS2, PS3, S1) and the one characterized lineage of *P. lutzii* (Pb01-like) can infect humans, and these groups can vary in virulence and induce different immune responses by the host [3], [4]. To better understand the pathogenesis and to enable genomics-based studies, the genomes of *Paracoccidioides* spp. were sequenced, analyzed and made publicly available in 2011 [1]. The Broad Institute of MIT and Harvard in partnership with the *Paracoccidioides* research community selected three reference isolates for sequencing and genomic analysis; assembly size for these strains varied between 29.1 and 32.9 Mb, and between 7,875 and 9,132 genes were identified in each strain [1]. These included two strains of *P. brasiliensis* (Pb18 representing the S1 lineage and Pb03 representing the PS2 lineage) and one strain of *P. lutzii* (Pb01) [1].

These sequenced isolates are extensively referenced in molecular biology and experimental mycology laboratories working with *Paracoccidioides* spp. and also other pathogenic fungi, including those working with yeast phase specific genes expressed during host infection. These sequences also serve as a reference to analyze high-throughput data increasingly generated by genomic, metagenomic, transcriptomic and proteomic approaches. Additionally, accurate sequences are critical for evolutionary analyses, e.g., to identify positively selected genes, as well as to provide new targets for the design of diagnostic assays.

The *P. brasiliensis* Pb18 and Pb03 strains and the *P. lutzii* Pb01 strain were sequenced using the sequencing technology and computational methods available at the time, which produced high quality draft assemblies. However, the assemblies included a large number of gaps and uncertain or low quality nucleotides in the final consensus sequences. Also, the annotation pipelines flagged only the most extreme annotation errors for curation and did not address the larger number of smaller scale errors in the gene models and underlying sequence [5]. Correction of such errors requires re-evaluation of the assembly consensus sequence and associated annotation.

Assembly errors that could not be detected in previous data and passed standard quality control criteria at that time can now be corrected using next generation sequencing (NGS) for systematic assembly improvement. These include errors in gene-containing regions of the original genomic assembly affected by poor quality sequence or ambiguities, which can cause incorrect gene structure predictions. Since predicted genes of reference genomes are now frequently used for homology-based inference or confirmation of gene structures in closely related species, errors in the reference sequence may be propagated to other genomes [6], [7]. Therefore, systematic improvement of a genome assembly and annotation can impact not just the understanding of that particular species, but also that of other related species for which it is used as a reference for comparison.

Here, we present an update of the three *Paracoccidioides* reference genome sequences achieved using Illumina re-sequencing to correct assembly errors and document the improvements obtained. The improved and updated reference genome assemblies and annotations of this important human fungal pathogen now allow more accurate SNP analyses, genome-wide evolutionary (e.g., selection) analyses that depend on high-quality sequences, phylogenetic footprinting studies of regulatory regions, or primer and probe design for diagnostic assays.

## Methods

### *Paracoccidioides* reference strains and previous sequencing

Three reference isolates of *Paracoccidioides* spp. (Pb01, Pb03 and Pb18), representing two species, were previously sequenced. The isolate of *P. lutzii* (Pb01) was a clinical isolate originating from an acute form of paracoccidioidomycosis (PCM) in an adult male. The two *P. brasiliensis* isolates were from individuals presenting chronic PCM; Pb03 represents the PS2 phylogenetic group and Pb18 the S1 group [1], [4]. In a partnership between the Broad Institute and the *Paracoccidioides* research community, these genomes were previously sequenced using multiple whole genome shotgun libraries constructed from genomic DNA for each strain; paired-end sequences were generated for each with Sanger technology and assembled using Arachne [1] (assembly v1; S1 Table).

### Re-sequencing of *Paracoccidioides* reference strains

The reference strains Pb01 (previously sequenced DNA sample) and Pb03 and Pb18 (newly extracted DNA samples) were re-sequenced using Illumina technology. For library construction, 100 ng of genomic DNA was sheared to ∼250 bp using a Covaris LE instrument and prepared for sequencing as previously described [8]. A library for each of the three samples was used to generate 101 base paired-end reads on the Illumina HiSeq2000 platform, producing an average genome coverage of 165X.

### Assembly improvement using Pilon

**Figure 1:**
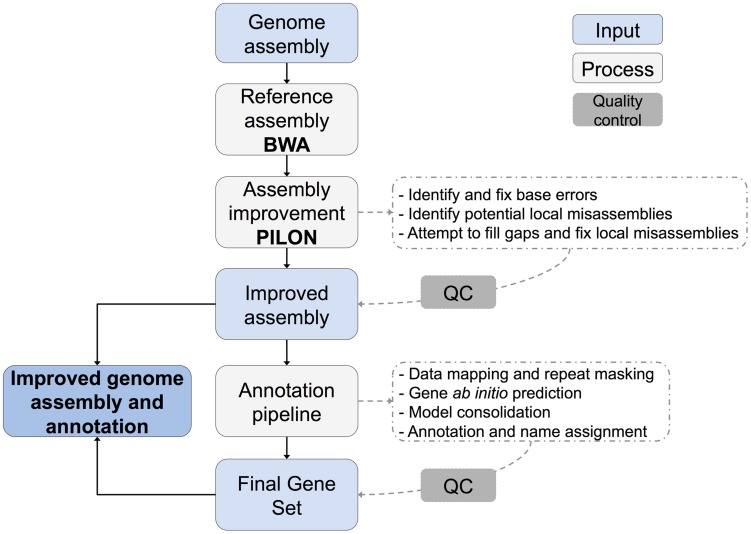
Overview of genome assembly and annotation improvement process.

To improve the genome sequence of *Paracoccidioides* spp. strains Pb01, Pb03 and Pb18, Illumina paired-end reads were aligned to the draft reference assemblies (assembly v1) using BWA version 0.5.9 with default settings [9]. The assembly consensus sequence was re-evaluated by providing these alignments as input to the automated assembly improvement program Pilon (version 1.4, default parameters, www.broadinstitute.org/software/pilon/). Pilon uses the Illumina read alignments for multiple classes of assembly correction. First, Pilon scans the read alignments for positions where the sequencing data disagree with the input genome (assembly v1) and corrects small errors such as single nucleotide differences and small insertion/deletion events. Second, Pilon looks for coverage and alignment discrepancies to identify potential mis-assemblies and larger variants. Finally, Pilon uses reads anchored adjacent to discrepant regions and gaps in the input genome to reassemble the region, attempting to fill in the true sequence including large insertions. As output, Pilon provides the sequence of this improved genome assembly (assembly v2; Fig. 1) along with files summarizing the changes and quality measures used in the assessment.

### Gene prediction and annotation

Protein-coding genes were predicted in the improved assemblies (assembly v2) using a combination of gene models from the prediction programs Augustus [10], Genemark-ES [11], GlimmerHMM [12], Genewise [13], and Snap [14], as well as automated revision based on EST data (e.g., from [15]) and manual gene revision of flagged calls. The predicted gene sets were then provided as input to EvidenceModeler (EVM) [16] to obtain the best consensus model for a given locus. The consistency of the gene models was evaluated by examining alignments of protein orthology groups identified using OrthoMCL [17]. EVMLite was used to rescue orphan genes not captured in EVM; only those genes with additional evidence such as overlap to Genewise or non-repeat HMMER3 PFAM domains were rescued, as well as non-redundant genes overlapping the OrthoMCL genes in clusters containing 2 or more genomes. Lastly spurious gene models matching repetitive or low-complexity sequences were removed.

For each *Paracoccidioides* genome, we compared the original annotation (v1) with the updated annotation (v2) to evaluate the changes in the new gene sets. To precisely characterize the types of changes across the v1 and v2 annotations, we first mapped the corresponding gene between the two assemblies. The v1 and v2 assemblies were aligned using nucmer [18], and the alignment coordinates were used to assign gene correspondence between the initial annotation v1 and the new annotation v2. This mapping also allowed us to preserve locus numbers in the updated gene set. Each annotated gene was assigned a locus number, keeping where appropriate the previous locus number of the form PAAG_##### (Pb01), PABG_##### (Pb03) or PADG_##### (Pb18), which serves as a unique identifier within each genome and across assemblies. New genes, merged genes, and genes with large structure and sequence changes in transcripts were assigned new and unique locus numbers following the last locus number of annotation v1. Locus numbers of deleted genes do not appear in the final gene sets.

### Evaluation of gene annotation improvements between *Paracoccidioides* strains

To evaluate whether the changes in gene sequence and structure produced a more accurate gene set, the gene sequences of annotation v1 and of the updated annotation v2 were compared via sequence similarity and orthology analysis. To evaluate the consistency of gene structures for orthologs as well as their conservation between species, OrthoMCL version 1.4 with a Markov inflation index of 1.5 and a maximum e-value of 1e-5 was used to identify orthologous clusters across the six total protein sets corresponding to annotations v1 and v2 of each *Paracoccidioides* strain. For each cluster group representing putative orthologs, we compared the maximum length difference among the three *Paracoccidioides* genes in annotation v2 to that in the annotation v1.

To compare the functional content of the v1 and v2 gene sets, we evaluated both protein domain families (PFAM) and pathway information (KEGG). Using HMMER3 [19], we mapped v27 of the PFAM domain database [20] to both the v1 and v2 gene sets. KEGG domains [21] from release 65 were also mapped to both gene sets using BLAST.

To evaluate changes in the gene structure, the corresponding transcripts from annotation v1 and v2 were identified as described above. We also aligned gene sets v1 and v2 using BLASTn [22] version 2.2.28+ with default parameters, using an in house Perl script to determine the types of modification for each gene, which included changes in gene length, gene coverage and percent nucleotide identity. We manually checked a random gene sample of each type of change (up to 10 genes) from both gene correspondence and BLAST analyses to verify that changes in gene set v2 were actually gene improvements. To evaluate changes in the coding regions of genes of high interest to the community, we selected known specific yeast-phase genes or virulence factors of *Paracoccidioides* spp., as well as other genes that are generally considered relevant for research on *Paracoccidioides* or related dimorphic pathogens, for manual review. The sequences of these genes' coding regions were aligned at the protein level with CLUSTALW [23] version 2.1, using both the v1 and v2 annotations.

### Gene annotation improvements using genes from CEGMA and from related dimorphic pathogenic fungi

The coverage of Core Eukaryotic Genes defined by CEGMA [24] was evaluated using the CoreAlyze tool (http://sourceforge.net/projects/corealyze/) to summarize results for all the v1 and v2 gene sets. BLASTp version 2.2.28+ was run with default settings using protein sets from annotations v1 and v2 as the database, with *Saccharomyces cerevisiae* and *Schizosaccharomyces pombe* CEGMA proteins as the query. We also included the protein gene sets of two close relatives of *Paracoccidioides*, the dimorphic fungal pathogens *Blastomyces dermatitidis* and *Histoplasma capsulatum*. In order to obtain a detailed picture of the changes where gene annotations were modified but not completely overridden, we compared protein sequences between the two versions, excluding proteins that were added or deleted from the final gene set v2, as well as proteins for which the new annotation was for a completely different transcript at the same locus. For a hit to be counted, the protein needed to match a protein in the reference set with at least 75% identity for the v1 and v2 annotations. This percent identity cutoff was determined empirically to eliminate spurious low similarity alignments. The percent identity and the bit score between the query protein and each version of the *Paracoccidioides* annotations (v1 and v2) were compared.

## Results

### Genome resequencing with NGS technology

The strains of the genomes of *Paracoccidioides* spp. previously sequenced [1], Pb18 and Pb03 and Pb01, were re-sequenced using Illumina 101 bp paired-end reads. This sequencing generated 93.6 million reads for Pb18 with an average coverage of 198X, 124.2 million reads for Pb03 with an average coverage of 150X and 110.0 million reads for Pb01 with an average coverage of 148X. This high coverage sequence data was then used to refine the consensus sequence of the original assembly by assessing differences between the new sequence and the previous assemblies. This can target a wide range of improvements, including correcting base calls, resolving ambiguous bases and closing gaps within scaffolds.

### Genome assembly improvement using Pilon

Fig. 1 shows a simplified overview of the workflow of genome improvement. The new Illumina data were used to systematically improve the three *Paracoccidioides* spp. assemblies using Pilon (http://www.broadinstitute.org/software/pilon/). Pilon bases its improvement calls on an alignment of the reference genome and the sequenced reads. The aligned bases and depth at each sequenced position provides evidence for the reference base or for an alternative; where changes are supported they can result in single base differences, insertion or deletion of single bases or larger regions, identification of collapsed regions and more complex changes and gap filling based on local reassembly. Reads of each of the genomes of Pb18, Pb03 and Pb01 were aligned to the corresponding reference assembly using BWA [9] and the resulting bam file was used as input for Pilon.

In each of the *Paracoccidioides* assemblies, Pilon identified and fixed base errors in the consensus sequence. The statistical improvements for the assemblies v2 of *Paracoccidioides* spp. are summarized in Table 1. The most frequent class of changes was single base substitutions, identified as single nucleotide polymorphisms (SNPs) between the assembly and reads. Between 3,018 and 3,290 single base errors were corrected in each assembly. Small insertions and deletions were also incorporated into each assembly. The major classes of changes can be attributed to bases added and removed in reassembly fixes, collapsed bases in the new assembly and the closing of gaps (Table 1). Regions of misassembly identified and fixed by Pilon resulted in bases added or removed but no new gaps opened. Across all the assemblies, 20% of all initial gaps were closed by Pilon; the number of gaps closed were 113, 56 and 212, for Pb18, Pb03 and Pb01, respectively. Overall, the assembly improvement process led to an increase of contig N50 for all strains. About ∼99% of low quality nucleotides in assemblies v1 were well supported or fixed with high flag coverage in assemblies v2.

**Table 1:** Summary of assembly metrics after Pilon improvement.

Pilon summary metrics	*P. brasiliensis*		*P. lutzii*
	Pb18	Pb03	Pb01
Read depth of coverage	127	146	148
SNPs	3,290	3,018	3,072
Ambiguous bases	246	222	221
Small insertion bases	957	1,083	1,062
Small deletion bases	725	714	628
Bases added in reassembly fixes	109,312	89,243	118,931
Bases removed in reassembly fixes	37,417	38,906	41,822
Gaps opened	0	0	0
Gaps closed	113	56	212
Collapsed regions	3	1	2
Collapsed bases	64,378	20,918	43,967
Increase in contig N50 (kb)	14.16	4.98	29.17

Overall, the *P. lutzii* Pb01 genome assembly was most substantially improved, based on comparing statistics for all v1 and v2 assemblies (S1 Table). The contig N50 for Pb01 v2 increased by 29.1 kb; more bases were added and removed after re-assembly fixes and more gaps were closed than in the other two genomes. The genome size and number of scaffolds of Pb18 and Pb03 were essentially unchanged. The Pb01 genome size decreased slightly from 32.94 to 32.93 Mb; the updated assembly contains one scaffold fewer, as two scaffolds were merged by closing the gap between them. The number of contigs was reduced in all three strains, which considering the increase in the contig N50 indicates that the assemblies v2 for Pb18, Pb03 and Pb01 were less fragmented. All these changes indicate that the genome assemblies v2 after Pilon improvements were more contiguous, contained more bases with high quality, and had fewer gaps and errors.

### Gene annotation improvement in updated assemblies

The gene annotations of the reference strains Pb18, Pb03 and Pb01 were updated using a pipeline to transfer and revise gene structures (Methods). The implemented annotation pipeline was an updated and improved version of the previous protocol used to annotate *Paracoccidioides* spp. assemblies v1. The current pipeline includes an updated set of gene prediction programs, including the EVM caller used to select the best call for each locus. Databases used for training these gene prediction programs are also more comprehensive, with more sequences available from the dimorphic fungi group for comparison. Also, the databases used for homology inference and functional annotation were updated since the previous annotation. In addition, we identified orthologs to evaluate the gene calls for consistency (see below). The incorporation of these new methods and data improved the evidence supporting gene prediction.

The updated gene sets are more consistent across the three *Paracoccidioides* genomes (S1 Table). The total gene count for the two *P. brasiliensis* genomes now only differs by 37 in v2 whereas the v1 gene counts differed by 866; overall the update removed 351 genes from Pb18 and added 552 genes to Pb03. *P. lutzii* (Pb01) also has a more similar gene count, due to 306 fewer genes in the v2 compared to v1. A more detailed view of the gene structure changes by major categories is provided in Table 2; these statistics were calculated by mapping the transcripts from the previous annotation to the corresponding locus on assembly v2. Notably, this analysis helped recover a large number of genes missed by the original annotation in each genome; the total genes newly added to a region was 840 in Pb18, 933 in Pb03 and 936 in Pb01. In addition, dubious genes were removed from each genome; the number of genes no longer present at the same locus was 1187 in Pb18, 490 in Pb03 and 1265 in Pb01. Other changes include extending or truncating transcripts, merging or splitting transcripts, changes to splice sites, and changes to UTRs (Table 2). Only 23% of genes in the v2 annotations were unchanged from v1; the primary transcripts were identical for 1816 genes in Pb18, 2599 in genes Pb03 and 1581 in genes Pb01. Genes with any type of change in their coding sequences represent a smaller subset, in total 5734 (68%) in Pb18, 4895 (58%) in Pb03 and 6309 (71%) in Pb01.

**Table 2:** Summary of annotation changes in protein coding genes.

Change type	*P. brasiliensis*		*P. lutzii*	Change description
	Pb18	Pb03	Pb01	
Add	840	933	936	Gene added to a region that previously had none
Splice site	1,124	1,122	1,000	Same start and same stop; internally, a splice site moved
Extended	3	6	2	Splice agreement, new model is longer; upstream start and downstream stop
Start Extended	262	329	307	Splice agreement and same stop; new model is longer, upstream start
Stop Extended	46	38	30	Splice agreement and same start; new model is longer, downstream stop
Shift	15	12	18	Splice agreement; new model has upstream, or downstream, start and stop
Truncated	5	4	2	Splice agreement, new model is shorter; downstream start and upstream stop
Start Truncated	237	208	276	Splice agreement and same stop; new model is shorter, downstream start
Stop Truncated	7	11	6	Splice agreement and same start; new model is shorter, upstream stop
UTR	1,504	802	1,679	Splice agreement and same start and same stop, but differ in UTR
Cluster	12	9	16	Multiple old genes map to multiple new genes; complex change
Merge	118	88	102	Multiple old genes have been merged into one
Split	402	509	495	Single old gene has been split into multiple new genes
Other CDS change	1,999	1,757	2,376	Other model not covered by another category or multiple models
None	1,816	2,599	1,581	Primary transcript is identical
Total	8,390	8,427	8,826	Total genes in current annotation version 2

**Figure 2:**
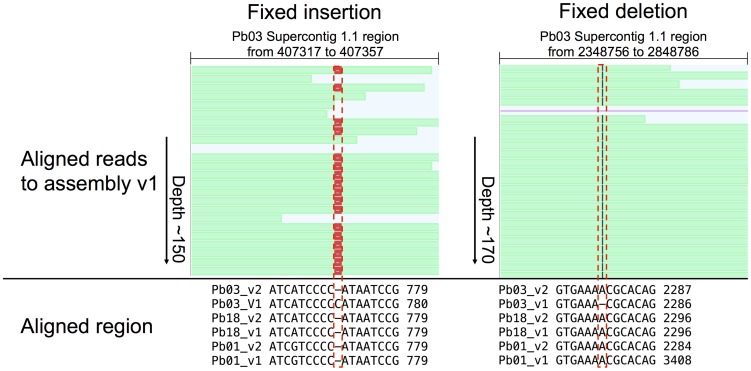
Examples of an artifactual insertion and an artifactual deletion that were corrected during the update of the *P. brasiliensis* Pb03 genome sequence. Screenshots of Pilon-generated genome browser tracks in GenomeView v1.0 [35] show the evidence used by Pilon to recognize and correct an incorrect insertion in the gene PABG_00120 (left) and an incorrect deletion in the gene PABG_00790 (right). Tracks (top panels) depict paired-end reads (green) aligned to the corresponding region of the reference assembly v1, a subset of the total depth of ∼150X or ∼170X; these alignments were used by Pilon to refine the consensus sequence, generating the improved Pb03 assembly v2. Positions in the v1 assembly where aligned reads suggest a change due to either a gap (red box) or an insertion (black line) are indicated with dashed red boxes. The changes suggested by Pilon are also supported by conservation of the changed bases in a multiple alignment (bottom panels) with the corresponding region of *P. brasiliensis* Pb18 and *P. lutzii* Pb01.

Both sequence addition (gap filling and local reassembly) and small changes in the genome assemblies (single-nucleotide substitutions or insertion/deletion events (indels)), contributed to the improvement of the gene annotation in the update v2. Two examples of how indel correction fixed gene structures are shown in Fig. 2. In the first case (left panel), an extra C was inserted at a polyC tract in PABG_00129 of Pb03; correction of this position resulted in extending the coding DNA sequence (CDS) of this gene by 423 bases. In the second case (right panel), an A was deleted at a polyA tract in PABG_00790; correction of this position also corrected the reading frame, allowing for removal of a false intron that was needed to step over a stop codon and extension of the CDS of this gene by 252 bases. While these are small changes to the underlying assembly, both have had larger impact on correcting these gene structures.

**Figure 3:**
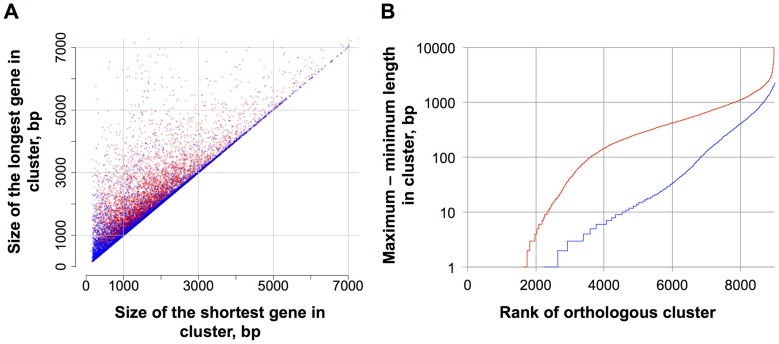
Improved consistency of gene annotation in v2 genomes. The final predicted gene sets of the three *Paracoccidioides* strains were clustered using OrthoMCL, in v1 and v2. The scatterplots (A) compare, for each clustered group, the maximum length versus the minimum length of the three *Paracoccidioides* genes in the same cluster, for each of the two versions. The scatterplot contrasts the maximum-minimum pairs from annotation v1 (red points) and those from annotation v2 (blue points). The location of blue points closer to the diagonal illustrates that the annotation v2 was more consistent across the three genomes with smaller differences in gene length. In the same sense, the rank plots (B) show the difference between maximum and minimum length for each clustered group, for each of the two versions; again annotation v2 (blue line) showed fewer (later increase) and smaller (more gradual increase) differences, corresponding to the improvement of the genome annotation in v2.

The annotation improvements were also analyzed by comparing the alignments of orthologs for all three *Paracoccidioides* genomes, identified by OrthoMCL (Methods). For orthologs identified either from the v1 or v2 assemblies, maximum and minimum gene length was computed for each ortholog cluster. In comparing these gene lengths (Fig. 3A), the v1 gene annotations (red points in scatterplot) exhibited a higher variation among Pb18, Pb03 and Pb01 orthologs compared to annotation v2 (blue points). The positions that are closer to the diagonal correspond to smaller differences in gene length between orthologs; as expected for an improved annotation, the v2 points are closer to the diagonal than v1. These differences between maximum and minimum length of the genes within each orthologous cluster group were also plotted on a logarithmic scale (Fig. 3B), based on sorting cluster differences from smallest to largest. The v2 annotation differences (blue curve) were lower and well separated from the v1 annotation (red curve), providing additional support of the increased length concordance in the v2 annotation.

Further analysis of gene conservation also supported the greater consistency among the *Paracoccidioides* spp. genomes in the v2 annotation. The number of genes found in all three genomes increased, whereas the number of unique genes specific to only one genome decreased; this has produced a more uniform set of protein coding genes (Figure S2). The improved structural annotation also led to improvements in functional annotation. The v2 annotation had more genes with assigned protein domain families (PFAM) and pathway information (KEGG), using the same version of these databases for the v1 and v2 gene sets (Figure S2). This supports the higher functional content of the revised gene sets, despite the lower total gene counts in two of the genomes.

**Figure 4:**
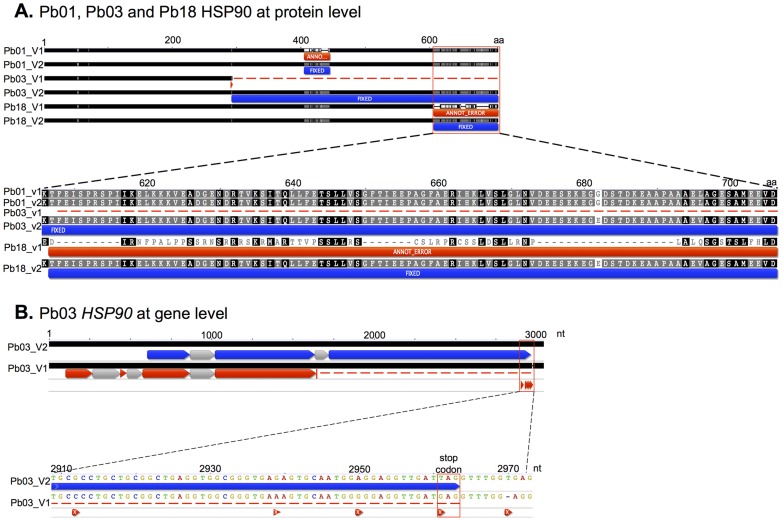
Diverse error correction for the 90 kDa heat shock protein (HSP90 gene) of *Paracoccidioides* spp. (A) In this example different annotation errors were present in v1 of all three *Paracoccidioides* reference strains, all of which were fixed in v2 after Pilon improvement and re-annotation. The example also illustrates how one or more single-nucleotide errors, unknown single nucleotides (N's), or single nucleotides that were erroneously reported as absent or duplicated by a Sanger sequencer can amplify across annotations, generating radically different gene structure (intron/exon and/or gene boundary) predictions. (B) Five changes are shown at assembly (DNA sequence) level, one of which was a single nucleotide error in a stop codon; as a result, the gene-calling program did not recognize the end of an exon and it was not reported.

We also manually reviewed and curated the predicted structures of a number of protein-coding genes that are of importance to the *Paracoccidioides* research community, including well-characterized yeast-phase specific genes and other virulence factors. This introduces changes to the transcript sequence of 27 of these genes (Table 3). The improvements to the assemblies resulted in updated transcript predictions for the vast majority of genes of the three reference strains, with substantially corrected gene structures for several virulence-associated or yeast-phase specific genes of central importance in *Paracoccidioides* or other dimorphic fungi, including *HSP90* [25], Pb*GP43* [26], Pb*P27* [27], *RYP1-3* [28], *BAD1* [29], catalase B, alpha 1,3 glucan synthase and the beta glucan synthase target gene *FKS1* [30], [31]. An extreme example is the *HSP90* gene, where corrections were made to the sequence of each of the three *Paracoccidioides* genomes (Fig. 4). This example illustrates the annotation errors in v1 of all *Paracoccidioides* reference strains that were fixed in v2 after Pilon improvement and re-annotation. In this case one or more single-nucleotide errors, unknown single nucleotides (N's), and/or single nucleotides that were erroneously reported as absent or duplicated by Sanger sequencing resulted in radically different predicted gene structures (intron/exon and/or gene boundary errors). This is shown in detail for a cluster of errors present at the end of HSP90 in Pb03 (Fig. 4B), which included alteration of the proper stop codon, resulting in premature truncation of this gene. Another example of coding sequence updates to multiple genomes is shown for FKS1, where different regions of the Pb03 and Pb18 proteins were restored in the updated assemblies and annotations (Figure S1).

**Table 3:** Changes in updated annotations of known yeast-phase specific genes or virulence factors of *Paracoccidioides* and other dimorphic human pathogenic fungi.

Gene name description	Annotation v2 IDs			Type of change			Ref.
	*P. lutzii*	*P. brasiliensis*		*P. lutzii*	*P. brasiliensis*		
	Pb01	Pb03	Pb18	Pb01	Pb03	Pb18	
	PAAG_	PABG_	PADG_	PAAG_	PABG_	PADG_	
1,3-beta-glucan synthase component GLS1 (*FKS1*)	05071	04524	11846*	None	Splice site	CDS/ID	[36]
1,3-beta-glucanosyltransferase (*GEL1*)	03782	00831	03286	Splice site	Splice site	Splice site	[31]
4-hydroxyphenylpyruvate dioxygenase (*4-HPPD*)	02615	03102	01636	UTR	Splice site	UTR	[37]
Alpha-1,3-glucan synthase (*AGS1*)	03297	00726	03169	CDS	CDS	Splice site	[38], [39]
Alternative oxidase (*AOX*)	01078	01661	03747	UTR	UTR	UTR	[40], [41]
bZIP transcription factor (*HAPX*)	04257	04038	07492	UTR	UTR	UTR	[42], [43]
Catalase B (*CATB*)	01553	03611	00225	None	None	Stop extended	[44], [45]
Catalase peroxixomal (*CATP*)	01454	01943	00324	Stop extended	Start truncated	Start truncated	[46]
Conserved hypothetical protein (*P27*)	08096	07332	08402	UTR	None	UTR	[27]
Cu Zu superoxide dismutase *(SOD1*)	04164	03954	07418	CDS	CDS	CDS	[47], [48]
Cu Zu superoxide dismutase *(SOD3*)	02971	00431	02842	UTR	None	None	[36], [47]
Dimorphism regulator histidine kinase (*DRK1*)	05810	06372	07579	UTR	CDS	CDS	[49]
Glucan 1,3-beta-glucosidase (*GP43*)	05770	06340	07615	UTR	UTR	UTR	[26]
Glyceraldehyde-3-phosphate dehydrogenase *(GAPDH)*	08468	00022	02411	Splice site	Extended	UTR	[48], [50]
HAD-superfamily hydrolase (*HAD32*)	00503	06765	02181	UTR	Splice site	Splice site	[51]
Heat shock protein 60 Kda (*HSP60*)	08059	07300	08369	UTR	UTR	UTR	[36], [52]
Heat shock protein 90 Kda (*HSP90*)	05679	06249	07715	Splice site	CDS	CDS	[25]
L-ornithine 5-monooxygenase (*SID1*)	01682	03730	00097	Splice site	None	None	[43], [53]
Tubulin beta chain (*TUB*)	03031	00486	02900	CDS	CDS	CDS	[15]
Adhesin WI-1 (*BAD1*)	08980	07814	**12525°**	UTR	None	New gene	[29], [54]
cAMP-independent regulatory protein pac2 (*RYP1*)	11652**	06919	06243	CDS/ID	None	UTR	[28], [55]
Ornithine decarboxylase (*ODC*)	03153	00600	03032	None	None	UTR	[56]
Required for yeast phase growth 2 (*RYP2*)	02671	03151	11302***	Splice site	Splice site	CDS/ID	[28], [57]
Required for yeast phase growth 3 (*RYP3*)	06081	06575	08037	Start extended	CDS	Stop extended	[28], [57]
Urease	00954	01291	03871	CDS	Start truncated	CDS	[58]
Urease accessory protein (*UREG)*	06237	05255	07010	UTR	Start truncated	UTR	[59]
Ureidoglycolate hydrolase (*UGH*)	04751	00102	02493	UTR	UTR	UTR	[59]

*IDs from version 1 *PADG_04920; **PAAG_03579; ***PADG_01695.

°New gene that was not reported previously.

The improvements in the annotation v2 were also analyzed for completeness by comparing to a set of highly conserved fungal genes defined by CEGMA and to protein sets of related dimorphic human pathogenic fungi. Genes in the v2 annotation showed a higher coverage of both the CEGMA and related dimorphic fungal data sets in comparison with annotation v1, suggesting these v2 genes are more complete (Figure S3). Furthermore, we examined the level of conservation to other fungi, by analyzing the difference of the BLASTp score between the v1 and v2 protein sets compared to those of *B. dermatitidis*, *H. capsulatum* and the CEGMA genes of *S. cerevisiae* and *S. pombe*. We observed that in all cases the v2 annotation had more hits greater than the minimum-similarity cutoff, and that the vast majority of genes of the v2 annotation had higher BLAST score values than their counterparts from v1 annotation (Figure S4).

## Discussion

The initial draft genomes of three isolates of *Paracoccidioides* (*P. brasiliensis* isolates Pb03 and Pb18, and *P. lutzii* isolate Pb01) served as the first complete genome references for this fungal species [1]. Although these assemblies were obtained using the best technology available at that time, they included gaps and low quality sequence in genic and intergenic regions, which in turn resulted in a number of suboptimal gene structures, coding sequences and predicted protein sequences. This work has revised these reference genomes, providing the *Paracoccidioides* community with more complete and accurate sequences; this provides a more accurate foundation for future genome-based, molecular biological or genetic research on paracoccidioidomycosis and the fungal strains that cause it. The strategy we have followed will more widely be useful also for other groups wishing to update fungal and other microbial genomes in future.

The updating of a reference genome, in particular of the underlying assembly and annotation, can be thought of as a largely computational form of deep sequence curation. The success of the update we present here shows that next-generation sequencing (NGS) together with publicly available software tools can markedly enhance the quality of a eukaryotic genome resource. Indeed, the availability of affordable NGS sequencing opportunities makes such endeavors accessible to small bioinformatics groups. The massively computer-assisted component of such an update, which can include tabular and graphical views for monitoring improvements and performing quality control, can be complemented by choosing and following a few ‘guide genes’ to evaluate the process. This focused analysis provides tangible examples of how the update affected predicted properties of important genes, such as gene structure or encoded proteins.

The accuracy of a genome sequence and associated annotations are critically important for many types of analysis; therefore validating and improving the accuracy of the sequence and annotation can have wide impact, especially for methods highly sensitive to sequence errors. One example involves examining a genome sequence for evidence of genes and genic regions likely to be under positive selection. Such genes and genic regions, which are believed to be relatively rare in many eukaryotic genomes (see, e.g., [32]), are sometimes associated in pathogenic organisms with virulence or rapid adaptation to host conditions, including resistance to defense by the host or avoidance of the host immune system. An example of such adaptation has been found for surface proteins of diverse pathogens [32] and in fungi of the proline-rich antigen gene in *Coccidioides* spp. [33]. Positive selection can also occur in response to antimicrobial drugs, as in chloroquine resistance in *Plasmodium falciparum* [34]. Candidate regions under positive selection are commonly identified as sections of coding regions having unusually high rates of nonsynonymous (amino-acid changing) substitutions. Precisely because such regions are quite rare, a coding region of low sequence quality having several sequencing errors could be categorized as a region under positive selection, and if there are several such regions in a genome, an automated genome-wide screen will report a high percentage of false positives. Conversely, assembly and annotation improvements such as we describe here can effectively evaluate and fix such regions of a genome so that even error-sensitive evolutionary analyses become realistic.

Similar considerations also apply to analyses that have more obvious clinical relevance. For example, improving a DNA sequence's accuracy can bring it closer to being ‘clinical grade’ or ‘diagnostic grade’. Indeed, identification of a clinical sample of a human pathogenic fungus isolated in a hospital using sequence comparison requires certainty that any nucleotide differences (e.g., resulting from single nucleotide polymorphisms/SNPs) observed between the sequenced sample and trusted reference strain(s) or isolate(s) are not simply errors in the reference. For fungi encountered in clinical contexts, only one or a few traditionally used loci are typically represented by reliable reference sequences, which are often from the ribosomal DNA, or from one or two protein-coding genes known beforehand to be diagnostically informative. Reference diagnostics, as well as diagnostic PCR assays (e.g., primer/probe design in real-time PCR assays), depend on such regions that have been reliably characterized at the molecular level in a fair number of related species or strains that could be present in clinical settings. Whole-genome gene sets offer, however, new perspectives; if their sequence quality is high, one could then systematically and exhaustively screen alignments of the full gene sets for diagnostically promising genes and genic regions that are likely to be informative for the identification task at hand. Such genome-wide screens should be able to identify new, candidate target loci, and molecular assays could then be developed for them and validated. Genome sequences also allow for metagenomic or metatranscriptomic analysis, where reference genomes enable identification of the pool random sequence from the population of microbes in a sample. Such wide applications will be better powered by efforts such as this to improve the set of reference genomes that form a fundamental basis of comparison and analysis.

By re-sequencing three reference strains of *Paracoccidioides* spp., using deep sequencing depth of Illumina paired-end reads, we have been able to substantially improve the assemblies and annotations for this important human fungal pathogen. Here we have presented the updated and improved annotated genome sequences, which constitute new references that can be used in diverse future molecular projects by those working in the field of medical mycology. Since the process leading to the new sequences is largely automated using publicly available programs and the NGS technology used is cost-effective, the success of our strategy represents a proof of concept that may stimulate similar updates of other genomes in future.

## Supporting Information

Figure S1 **Regions of FKS1 protein sequence alignment highlighting changes between genome versions.** This example is of a gene relevant to medical and experimental mycology, the 1,3-beta glucan synthase component *FKS1*. The colored regions (blue text for bases, red ‘-‘ for gaps in the alignment) illustrate the improvements in the annotation (‘CDS’ category) that led to improved protein sequences in two of the three *Paracoccidioides* strains.
(TIF)

Figure S2 **Comparison of ortholog conservation and annotation in v1 and v2 genomes.** The final gene sets of each annotation version were clustered using OrthoMCL. (**A**) Bar chart showing the relative contributions of core, auxiliary and unique genes to the final gene clusters of versions 1 and 2. The clustered groups were categorized as ‘core’ if present in all strains, ‘aux’ if present in two strains and ‘uniq’ if present in one strain. (**B**) Venn diagram showing numbers of shared and unique genes in annotation v2. (**C**) Total numbers of predicted genes, and total numbers of predicted genes that were assigned functional annotation from PFAM and/or KEGG. In all cases, the annotation v2 is more consistent or homogeneous across the three strains than annotation v1: there are more core genes in annotation v2 and also in v2 the two strains Pb03 and Pb18 from the same species (*P. brasiliensis*) give more similar results (bar charts). Furthermore, the new annotation has more genes with assigned functional annotation even using the same version of the databases.
(TIF)

Figure S3 **Coverage of Core Eukaryotic Genes (CEGs) in original and updated genomes.** More genes in annotation v2 had higher percent of coverage of CEGs in comparison with annotation v1. This analysis was performed and plotted using the CoreAlyze tool (http://sourceforge.net/project/corealyze).
(TIF)

Figure S4 **Difference in BLAST scores using the protein sets of** ***Paracoccidioides*** **annotations v1 and v2.** The references used for the BLAST matching were the protein sets of two dimorphic pathogenic fungi that are closely related to *Paracoccidioides* (top row) and the Core Eukaryotic genes of the fungi in CEGMA (bottom row). The comparison shows that the vast majority of proteins with any change, included in the comparison, have higher BLAST score in annotation v2. Here the graphs depict the results for the Pb03 strain; Pb18 and Pb01 showed the same pattern. The horizontal axis shows the genes numbered in order of increasing score difference (v2 minus v1).
(TIF)

Table S1 **Summary of assembly and gene statistics of v1 and the updated v2 of the three reference genomes of *Paracoccidioides* spp.**
(XLSX)

## References

[1] Desjardins CA, Champion MD, Holder JW, Muszewska A, Goldberg J, et al (2011) Comparative genomic analysis of human fungal pathogens causing paracoccidioidomycosis. PLoS Genet 7: e1002345 10.1371/journal.pgen.1002345 22046142 PMC3203195

[2] Brummer E, Castaneda E, Restrepo A (1993) Paracoccidioidomycosis: an update. Clin Microbiol Rev 6: 89–117. 8472249 10.1128/cmr.6.2.89 PMC358272

[3] Theodoro RC, Teixeira M de M, Felipe MSS, Paduan KDS, Ribolla PM, et al (2012) Genus paracoccidioides: Species recognition and biogeographic aspects. PloS One 7: e37694 10.1371/journal.pone.0037694 22666382 PMC3364295

[4] Matute DR, McEwen JG, Puccia R, Montes BA, San-Blas G, et al (2006) Cryptic speciation and recombination in the fungus *Paracoccidioides brasiliensis* as revealed by gene genealogies. Mol Biol Evol 23: 65–73 10.1093/molbev/msj008 16151188

[5] Haas BJ, Zeng Q, Pearson MD, Cuomo CA, Wortman JR (2011) Approaches to Fungal Genome Annotation. Mycology 2: 118–141 10.1080/21501203.2011.606851 22059117 PMC3207268

[6] Muñoz JF, Gallo JE, Misas E, McEwen JG, Clay OK (2013) The eukaryotic genome, its reads, and the unfinished assembly. FEBS Lett 587: 2090–2093 10.1016/j.febslet.2013.05.048 23727201

[7] Cruveiller S, Jabbari K, Clay O, Bemardi G (2003) Compositional features of eukaryotic genomes for checking predicted genes. Brief Bioinform 4: 43–52. 12715833 10.1093/bib/4.1.43

[8] Fisher S, Barry A, Abreu J, Minie B, Nolan J, et al (2011) A scalable, fully automated process for construction of sequence-ready human exome targeted capture libraries. Genome Biol 12: R1 10.1186/gb-2011-12-1-r1 21205303 PMC3091298

[9] Li H, Durbin R (2010) Fast and accurate long-read alignment with Burrows-Wheeler transform. Bioinformatics 26: 589–595 10.1093/bioinformatics/btp698 20080505 PMC2828108

[10] Stanke M, Waack S (2003) Gene prediction with a hidden Markov model and a new intron submodel. Bioinforma Oxf Engl 19 Suppl 2ii215–ii225. 10.1093/bioinformatics/btg1080 14534192

[11] Ter-Hovhannisyan V, Lomsadze A, Chernoff YO, Borodovsky M (2008) Gene prediction in novel fungal genomes using an ab initio algorithm with unsupervised training. Genome Res 18: 1979–1990 10.1101/gr.081612.108 18757608 PMC2593577

[12] Majoros WH, Pertea M, Salzberg SL (2004) TigrScan and GlimmerHMM: two open source ab initio eukaryotic gene-finders. Bioinforma Oxf Engl 20: 2878–2879 10.1093/bioinformatics/bth315 15145805

[13] Birney E, Clamp M, Durbin R (2004) GeneWise and Genomewise. Genome Res 14: 988–995 10.1101/gr.1865504 15123596 PMC479130

[14] Korf I (2004) Gene finding in novel genomes. BMC Bioinformatics 5: 59 10.1186/1471-2105-5-59 15144565 PMC421630

[15] Goldman GH, dos Reis Marques E, Duarte Ribeiro DC, de Souza Bernardes LA, Quiapin AC, et al (2003) Expressed sequence tag analysis of the human pathogen *Paracoccidioides brasiliensis* yeast phase: identification of putative homologues of *Candida albicans* virulence and pathogenicity genes. Eukaryot Cell 2: 34–48. 12582121 10.1128/EC.2.1.34-48.2003 PMC141168

[16] Haas BJ, Salzberg SL, Zhu W, Pertea M, Allen JE, et al (2008) Automated eukaryotic gene structure annotation using EVidenceModeler and the Program to Assemble Spliced Alignments. Genome Biol 9: R7 10.1186/gb-2008-9-1-r7 18190707 PMC2395244

[17] Li L, Stoeckert CJ, Roos DS (2003) OrthoMCL: identification of ortholog groups for eukaryotic genomes. Genome Res 13: 2178–2189. 12952885 10.1101/gr.1224503 PMC403725

[18] Kurtz S, Phillippy A, Delcher AL, Smoot M, Shumway M, et al (2004) Versatile and open software for comparing large genomes. Genome Biol 5: R12. 14759262 10.1186/gb-2004-5-2-r12 PMC395750

[19] Eddy SR (2011) Accelerated Profile HMM Searches. PLoS Comput Biol 7: e1002195 10.1371/journal.pcbi.1002195 22039361 PMC3197634

[20] Finn RD, Bateman A, Clements J, Coggill P, Eberhardt RY, et al (2014) Pfam: the protein families database. Nucleic Acids Res 42: D222–D230 10.1093/nar/gkt1223 24288371 PMC3965110

[21] Kanehisa M, Goto S (2000) KEGG: kyoto encyclopedia of genes and genomes. Nucleic Acids Res 28: 27–30. 10592173 10.1093/nar/28.1.27 PMC102409

[22] Altschul SF, Madden TL, Schaffer AA, Zhang J, Zhang Z, et al (1997) Gapped BLAST and PSI-BLAST: a new generation of protein database search programs. Nucleic Acids Res 25: 3389–3402. 9254694 10.1093/nar/25.17.3389 PMC146917

[23] Larkin MA, Blackshields G, Brown NP, Chenna R, McGettigan PA, et al (2007) Clustal W and Clustal X version 2.0. Bioinforma Oxf Engl 23: 2947–2948 10.1093/bioinformatics/btm404 17846036

[24] Parra G, Bradnam K, Korf I (2007) CEGMA: a pipeline to accurately annotate core genes in eukaryotic genomes. Bioinforma Oxf Engl 23: 1061–1067 10.1093/bioinformatics/btm071 17332020

[25] Tamayo D, Muñoz JF, Torres I, Almeida AJ, Restrepo A, et al (2013) Involvement of the 90 kDa heat shock protein during adaptation of *Paracoccidioides brasiliensis* to different environmental conditions. Fungal Genet Biol FG B 51: 34–41 10.1016/j.fgb.2012.11.005 23207691

[26] Torres I, Hernandez O, Tamayo D, Muñoz JF, Leitão NP, et al (2013) Inhibition of PbGP43 expression may suggest that gp43 is a virulence factor in *Paracoccidioides brasiliensis* . PloS One 8: e68434 10.1371/journal.pone.0068434 23874627 PMC3708949

[27] Torres I, Hernandez O, Tamayo D, Muñoz JF, García AM, et al (2014) *Paracoccidioides brasiliensis* PbP27 gene: knockdown procedures and functional characterization. FEMS Yeast Res 14: 270–280 10.1111/1567-1364.12099 24118983

[28] Beyhan S, Gutierrez M, Voorhies M, Sil A (2013) A temperature-responsive network links cell shape and virulence traits in a primary fungal pathogen. PLoS Biol 11: e1001614 10.1371/journal.pbio.1001614 23935449 PMC3720256

[29] Brandhorst TT, Wüthrich M, Warner T, Klein B (1999) Targeted gene disruption reveals an adhesin indispensable for pathogenicity of *Blastomyces dermatitidis* . J Exp Med 189: 1207–1216. 10209038 10.1084/jem.189.8.1207 PMC2193031

[30] Puccia R, Vallejo MC, Matsuo AL, Longo LVG (2011) The paracoccidioides cell wall: past and present layers toward understanding interaction with the host. Front Microbiol 2: 257 10.3389/fmicb.2011.00257 22194733 PMC3243086

[31] Rappleye CA, Goldman WE (2006) Defining virulence genes in the dimorphic fungi. Annu Rev Microbiol 60: 281–303 10.1146/annurev.micro.59.030804.121055 16753032

[32] Endo T, Ikeo K, Gojobori T (1996) Large-scale search for genes on which positive selection may operate. Mol Biol Evol 13: 685–690. 8676743 10.1093/oxfordjournals.molbev.a025629

[33] Johannesson H, Vidal P, Guarro J, Herr RA, Cole GT, et al (2004) Positive directional selection in the proline-rich antigen (PRA) gene among the human pathogenic fungi *Coccidioides immitis*, *C. posadasii* and their closest relatives. Mol Biol Evol 21: 1134–1145 10.1093/molbev/msh124 15034131

[34] Wootton JC, Feng X, Ferdig MT, Cooper RA, Mu J, et al (2002) Genetic diversity and chloroquine selective sweeps in Plasmodium falciparum. Nature 418: 320–323 10.1038/nature00813 12124623

[35] Abeel T, Van Parys T, Saeys Y, Galagan J, Van de Peer Y (2012) GenomeView: a next-generation genome browser. Nucleic Acids Res 40: e12 10.1093/nar/gkr995 22102585 PMC3258165

[36] Tavares AHFP, Silva SS, Dantas A, Campos EG, Andrade RV, et al (2007) Early transcriptional response of *Paracoccidioides brasiliensis* upon internalization by murine macrophages. Microbes Infect Inst Pasteur 9: 583–590 10.1016/j.micinf.2007.01.024 17387029

[37] Nunes LR, Costa de Oliveira R, Leite DB, da Silva VS, dos Reis Marques E, et al (2005) Transcriptome analysis of *Paracoccidioides brasiliensis* cells undergoing mycelium-to-yeast transition. Eukaryot Cell 4: 2115–2128 10.1128/EC.4.12.2115-2128.2005 16339729 PMC1317488

[38] Marques ER, Ferreira MES, Drummond RD, Felix JM, Menossi M, et al (2004) Identification of genes preferentially expressed in the pathogenic yeast phase of *Paracoccidioides brasiliensis*, using suppression subtraction hybridization and differential macroarray analysis. Mol Genet Genomics MGG 271: 667–677 10.1007/s00438-004-1016-6 15138890

[39] Rappleye CA, Engle JT, Goldman WE (2004) RNA interference in *Histoplasma capsulatum* demonstrates a role for alpha-(1,3)-glucan in virulence. Mol Microbiol 53: 153–165 10.1111/j.1365-2958.2004.04131.x 15225311

[40] Martins VP, Dinamarco TM, Soriani FM, Tudella VG, Oliveira SC, et al (2011) Involvement of an alternative oxidase in oxidative stress and mycelium-to-yeast differentiation in *Paracoccidioides brasiliensis* . Eukaryot Cell 10: 237–248 10.1128/EC.00194-10 21183691 PMC3067407

[41] Ruiz OH, Gonzalez A, Almeida AJ, Tamayo D, Garcia AM, et al (2011) Alternative oxidase mediates pathogen resistance in *Paracoccidioides brasiliensis* infection. PLoS Negl Trop Dis 5: e1353 10.1371/journal.pntd.0001353 22039556 PMC3201906

[42] Gauthier GM, Sullivan TD, Gallardo SS, Brandhorst TT, Vanden Wymelenberg AJ, et al (2010) SREB, a GATA transcription factor that directs disparate fates in *Blastomyces dermatitidis* including morphogenesis and siderophore biosynthesis. PLoS Pathog 6: e1000846 10.1371/journal.ppat.1000846 20368971 PMC2848559

[43] Hwang LH, Mayfield JA, Rine J, Sil A (2008) *Histoplasma* requires SID1, a member of an iron-regulated siderophore gene cluster, for host colonization. PLoS Pathog 4: e1000044 10.1371/journal.ppat.1000044 18404210 PMC2275787

[44] Holbrook ED, Smolnycki KA, Youseff BH, Rappleye CA (2013) Redundant catalases detoxify phagocyte reactive oxygen and facilitate *Histoplasma capsulatum* pathogenesis. Infect Immun 81: 2334–2346 10.1128/IAI.00173-13 23589579 PMC3697612

[45] Inglis DO, Voorhies M, Hocking Murray DR, Sil A (2013) Comparative transcriptomics of infectious spores from the fungal pathogen *Histoplasma capsulatum* reveals a core set of transcripts that specify infectious and pathogenic states. Eukaryot Cell 12: 828–852 10.1128/EC.00069-13 23563482 PMC3675998

[46] Chagas RF, Bailão AM, Pereira M, Winters MS, Smullian AG, et al (2008) The catalases of *Paracoccidioides brasiliensis* are differentially regulated: protein activity and transcript analysis. Fungal Genet Biol FG B 45: 1470–1478 10.1016/j.fgb.2008.08.007 18799136

[47] Youseff BH, Holbrook ED, Smolnycki KA, Rappleye CA (2012) Extracellular superoxide dismutase protects *Histoplasma* yeast cells from host-derived oxidative stress. PLoS Pathog 8: e1002713 10.1371/journal.ppat.1002713 22615571 PMC3355102

[48] Hernandez O, Garcia AM, Almeida AJ, Tamayo D, Gonzalez A, et al (2011) Gene expression during activation of *Paracoccidioides brasiliensis* conidia. Yeast Chichester Engl 28: 771–781 10.1002/yea.1902 21960298

[49] Nemecek JC, Wuthrich M, Klein BS (2006) Global control of dimorphism and virulence in fungi. Science 312: 583–588 10.1126/science.1124105 16645097

[50] Edwards JA, Chen C, Kemski MM, Hu J, Mitchell TK, et al (2013) *Histoplasma* yeast and mycelial transcriptomes reveal pathogenic-phase and lineage-specific gene expression profiles. BMC Genomics 14: 695 10.1186/1471-2164-14-695 24112604 PMC3852720

[51] Hernández O, Almeida AJ, Gonzalez A, Garcia AM, Tamayo D, et al (2010) A 32-kilodalton hydrolase plays an important role in *Paracoccidioides brasiliensis* adherence to host cells and influences pathogenicity. Infect Immun 78: 5280–5286 10.1128/IAI.00692-10 20876288 PMC2981316

[52] Guimarães AJ, Nakayasu ES, Sobreira TJP, Cordero RJB, Nimrichter L, et al (2011) *Histoplasma capsulatum* heat-shock 60 orchestrates the adaptation of the fungus to temperature stress. PloS One 6: e14660 10.1371/journal.pone.0014660 21347364 PMC3037374

[53] Parente AFA, Bailão AM, Borges CL, Parente JA, Magalhães AD, et al (2011) Proteomic analysis reveals that iron availability alters the metabolic status of the pathogenic fungus *Paracoccidioides brasiliensis* . PloS One 6: e22810 10.1371/journal.pone.0022810 21829521 PMC3145762

[54] Bohse ML, Woods JP (2007) RNA interference-mediated silencing of the YPS3 gene of *Histoplasma capsulatum* reveals virulence defects. Infect Immun 75: 2811–2817 10.1128/IAI.00304-07 17403872 PMC1932869

[55] Nguyen VQ, Sil A (2008) Temperature-induced switch to the pathogenic yeast form of *Histoplasma capsulatum* requires Ryp1, a conserved transcriptional regulator. Proc Natl Acad Sci U A 105: 4880–4885 10.1073/pnas.0710448105 PMC2290814 18339808

[56] Guevara-Olvera L, Hung CY, Yu JJ, Cole GT (2000) Sequence, expression and functional analysis of the *Coccidioides immitis* ODC (ornithine decarboxylase) gene. Gene 242: 437–448. 10721738 10.1016/s0378-1119(99)00496-5

[57] Webster RH, Sil A (2008) Conserved factors Ryp2 and Ryp3 control cell morphology and infectious spore formation in the fungal pathogen *Histoplasma capsulatum* . Proc Natl Acad Sci U A 105: 14573–14578 10.1073/pnas.0806221105 PMC2567189 18791067

[58] Mirbod-Donovan F, Schaller R, Hung C-Y, Xue J, Reichard U, et al (2006) Urease produced by *Coccidioides posadasii* contributes to the virulence of this respiratory pathogen. Infect Immun 74: 504–515 10.1128/IAI.74.1.504-515.2006 16369007 PMC1346605

[59] Whiston E, Zhang Wise H, Sharpton TJ, Jui G, Cole GT, et al (2012) Comparative transcriptomics of the saprobic and parasitic growth phases in *Coccidioides* spp. PloS One 7: e41034 10.1371/journal.pone.0041034 22911737 PMC3401177

