# Supplementary figures and images for: Genome Update of the Dimorphic Human Pathogenic Fungi Causing Paracoccidioidomycosis
Source: PLoS Negl Trop Dis. 2014 Dec 4;8(12):e3348. doi: 10.1371/journal.pntd.0003348 (PMC4256289; doi:10.1371/journal.pntd.0003348)

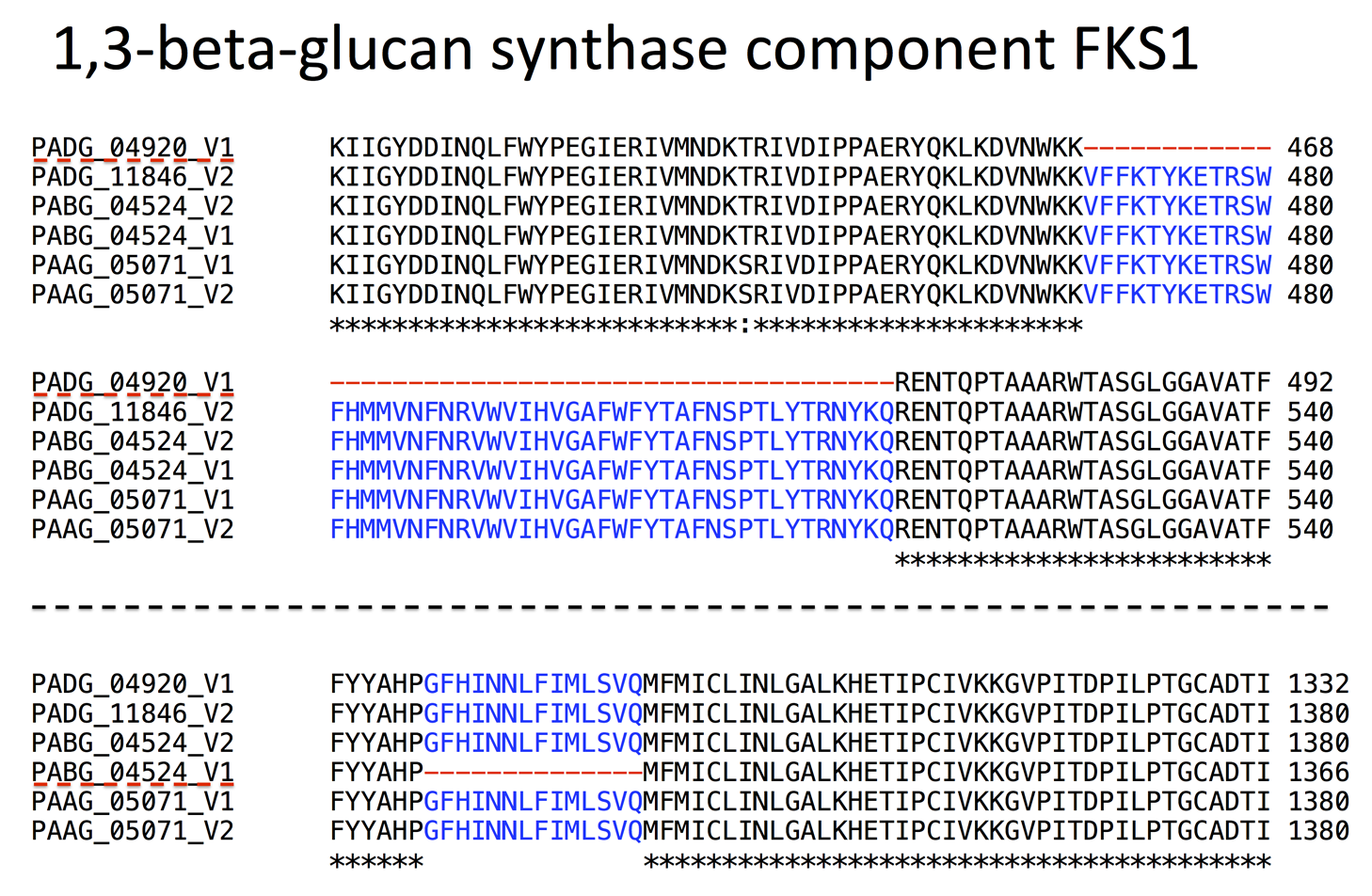

Supplement: Figure S1 — Regions of FKS1 protein sequence alignment highlighting changes between genome versions. This example is of a gene relevant to medical and experimental mycology, the 1,3-beta glucan synthase component FKS1. The colored regions (blue text for bases, red ‘-‘ for gaps in the alignment) illustrate the improvements in the annotation (‘CDS’ category) that led to improved protein sequences in two of the three Paracoccidioides strains. (TIF) [file pntd.0003348.s001.tif]

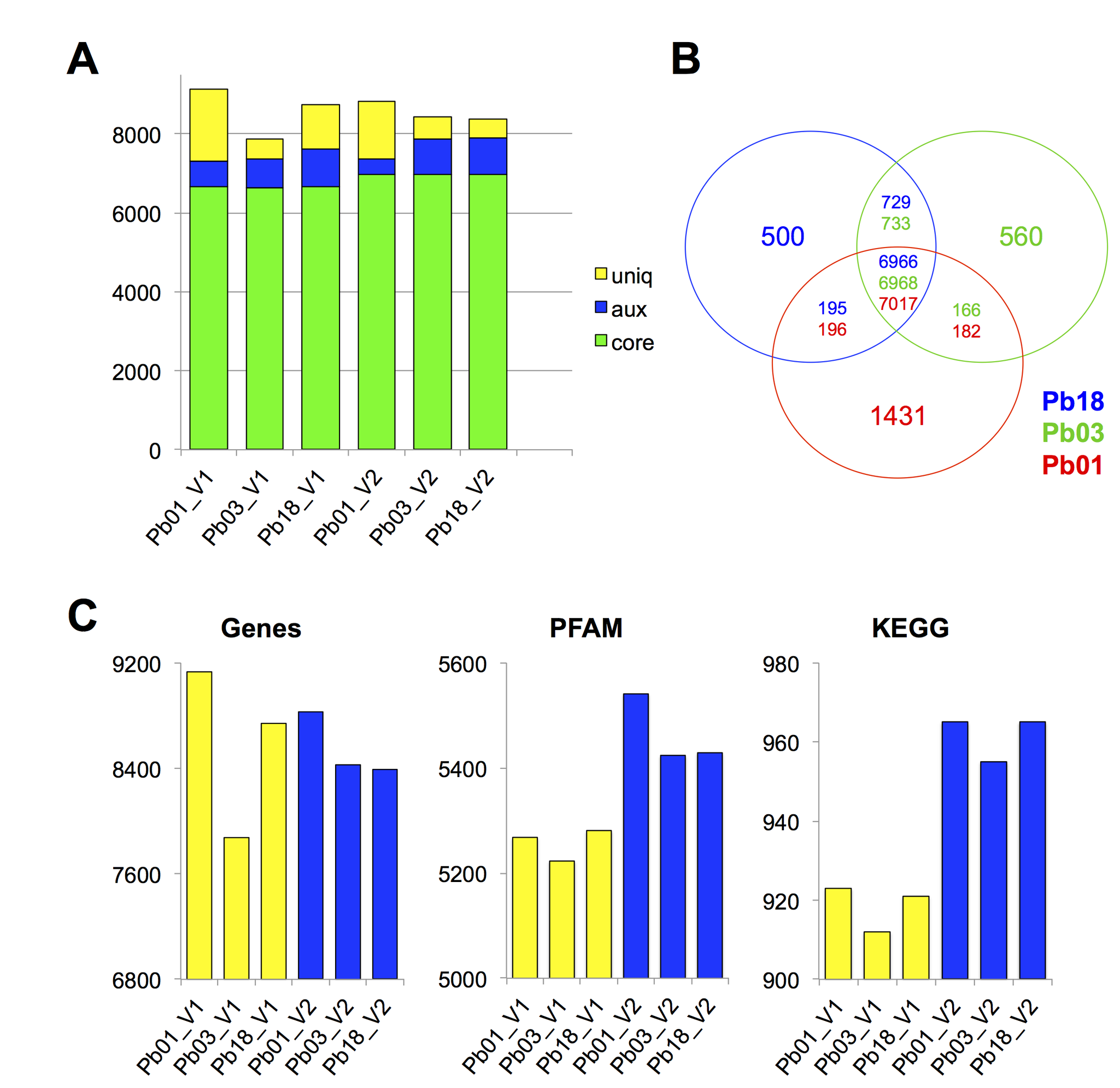

Supplement: Figure S2 — Comparison of ortholog conservation and annotation in v1 and v2 genomes. The final gene sets of each annotation version were clustered using OrthoMCL. (A) Bar chart showing the relative contributions of core, auxiliary and unique genes to the final gene clusters of versions 1 and 2. The clustered groups were categorized as ‘core’ if present in all strains, ‘aux’ if present in two strains and ‘uniq’ if present in one strain. (B) Venn diagram showing numbers of shared and unique genes in annotation v2. (C) Total numbers of predicted genes, and total numbers of predicted genes that were assigned functional annotation from PFAM and/or KEGG. In all cases, the annotation v2 is more consistent or homogeneous across the three strains than annotation v1: there are more core genes in annotation v2 and also in v2 the two strains Pb03 and Pb18 from the same species (P. brasiliensis) give more similar results (bar charts). Furthermore, the new annotation has more genes with assigned functional annotation even using the same version of the databases. (TIF) [file pntd.0003348.s002.tif]

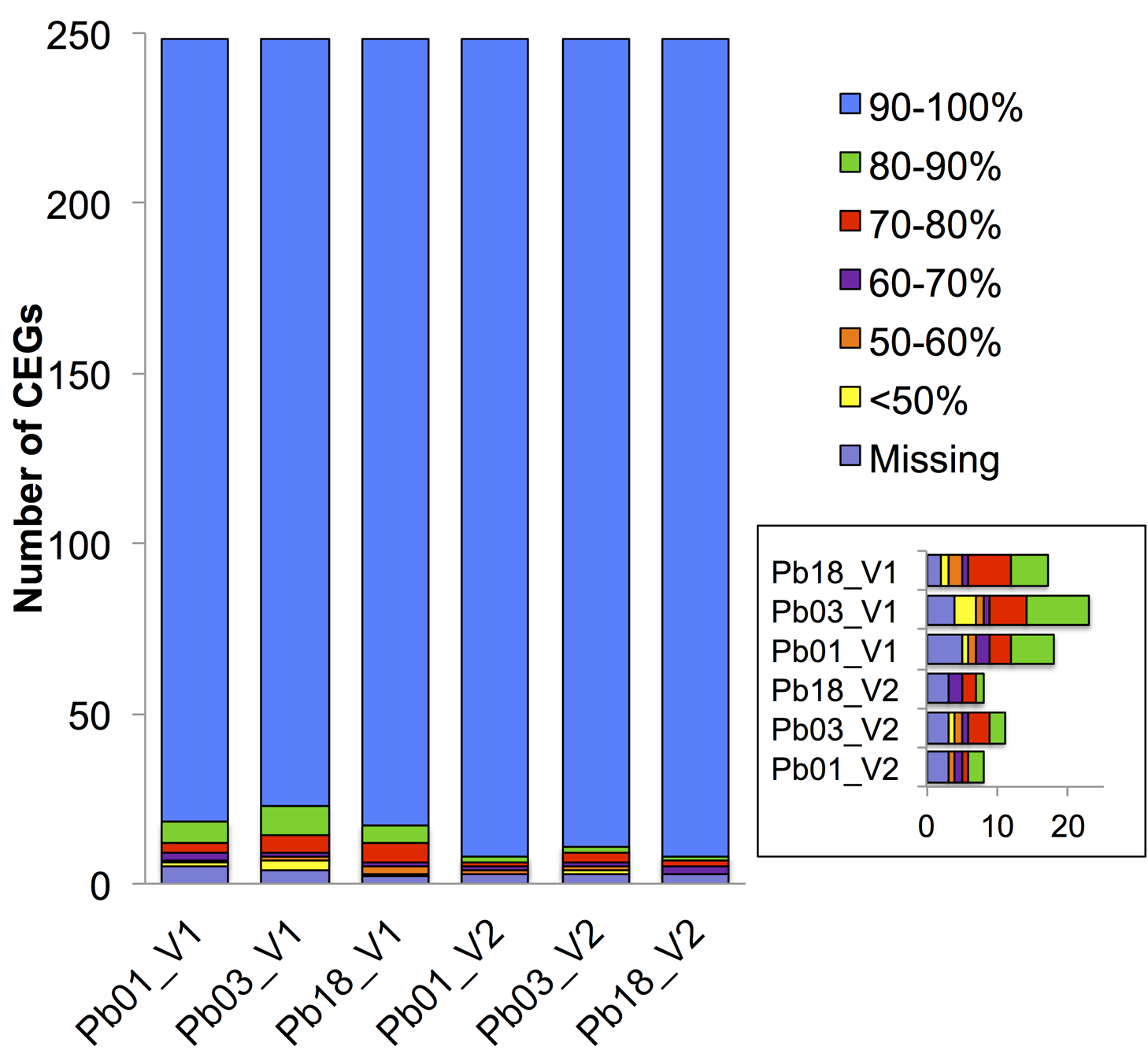

Supplement: Figure S3 — Coverage of Core Eukaryotic Genes (CEGs) in original and updated genomes. More genes in annotation v2 had higher percent of coverage of CEGs in comparison with annotation v1. This analysis was performed and plotted using the CoreAlyze tool (http://sourceforge.net/project/corealyze). (TIF) [file pntd.0003348.s003.tif]

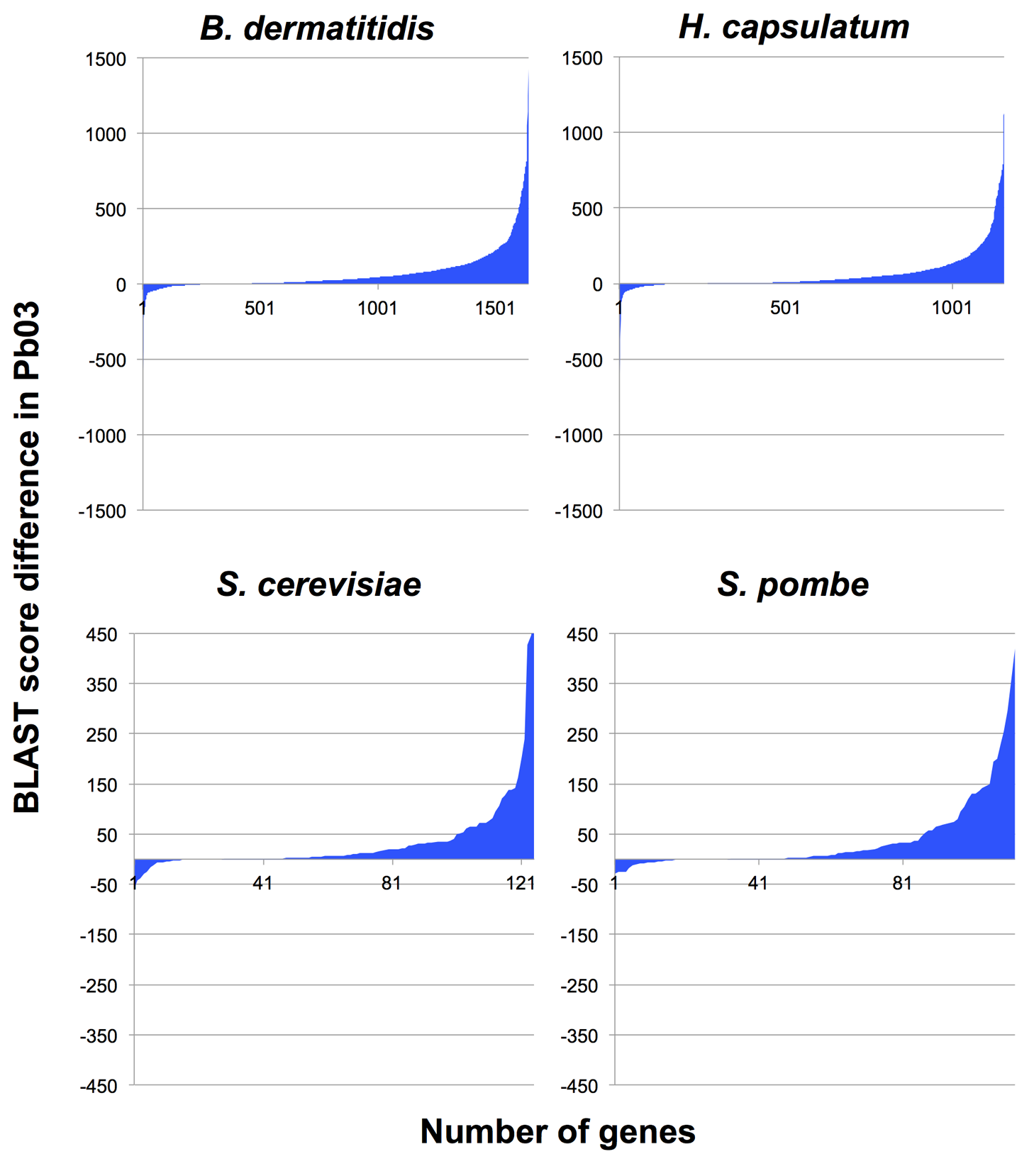

Supplement: Figure S4 — Difference in BLAST scores using the protein sets of Paracoccidioides annotations v1 and v2. The references used for the BLAST matching were the protein sets of two dimorphic pathogenic fungi that are closely related to Paracoccidioides (top row) and the Core Eukaryotic genes of the fungi in CEGMA (bottom row). The comparison shows that the vast majority of proteins with any change, included in the comparison, have higher BLAST score in annotation v2. Here the graphs depict the results for the Pb03 strain; Pb18 and Pb01 showed the same pattern. The horizontal axis shows the genes numbered in order of increasing score difference (v2 minus v1). (TIF) [file pntd.0003348.s004.tif]
